# Effect of Replacement of Fish Meal With Cottonseed and Blood Meals on Growth, Serum Biochemistry, Body Composition, and Antioxidant Capacity in GIFT Tilapia Juveniles (*Oreochromis niloticus*)

**DOI:** 10.1155/anu/5535909

**Published:** 2025-06-07

**Authors:** Xin Liu, Bo Liu, Qunlan Zhou, Cunxin Sun, Xiaochuan Zheng, Bo Liu

**Affiliations:** ^1^Wuxi Fisheries College, Nanjing Agricultural University, Wuxi 214081, China; ^2^Key Laboratory of Aquatic Animal Nutrition and Health, Freshwater Fisheries Research Center, Chinese Academy of Fishery Science, Wuxi 214081, China; ^3^Key Laboratory of Freshwater Fisheries and Germplasm Resources Utilization, Ministry of Agriculture and Rural Affairs, Freshwater Fisheries Research Center, Chinese Academy of Fishery Sciences, Wuxi 214081, China

**Keywords:** blood chemistry, fish meal replacement, growth metrics, proximate composition analysis

## Abstract

Nile tilapia of an initial average weight of 36.55 ± 0.15 g were stocked in 15 tanks at 20 fish per tank. Five isonitrogenous and isolipidic experimental diets (31.5% crude protein, 7.45% crude lipid) were formulated to replace 0% (D-0 diet), 25% (D-25 diet), 50% (D-50 diet), 75% (D-75), and 100% (D-100 diet) of fish meal (FM) with cottonseed meal (CSM) and blood meal (BM). After 60 days of rearing experiment, results showed that final body weight (FBW), specific growth rate (SGR), feed conversion ratio (FCR), weight gain rae (WGR), protein efficiency ratio (PER), the whole-body crude proteins and ash content exhibited no significant change at D-25, D-50, and D-75 group (*p* > 0.05) compared to the control. FBW, WGR, PER, SGR, crude protein, and ash were significantly lower, while FCR was significantly higher in D-100 compared to control (*p* < 0.05). Serum biochemical activities indicated that albumin (ALB), triglycerides (TGs), and total cholesterol (TC) exhibited no significant change (*p* > 0.05) among all treatments. However, glucose (GLU) concentration was significantly increased in the D-50 and D-75 groups compared with the control group. Meanwhile, alanine aminotransferase (ALT) was significantly lower in the D-100 group, aspartate transaminase (AST) was significantly higher in D-25, D-50, D-75, and D-100 groups, superoxide dismutase (SOD) activity was higher in D-75 group, and glutathione-peroxidase (GSH-Px) was lower in D-25 compared to the control group. These results suggest that D-75 is the optimal substitution level without adverse effects on GIFT tilapia juveniles.

## 1. Introduction

Fish is an important nutritional protein source for people, and it provides about 20% of animal protein for humans, according to the statistical data of FAO [1]. Feed is one of the important factors in the success of aquaculture and accounts for about 60%–70% of aquaculture's cost of production [2]. The dietary protein source is important for fish feeds. Fish meal (FM) is a major source of dietary protein in feed preparation [3]. With the reduced catch of wild fish due to overfishing in the sea has led to a reduction in FM production as well as an increase in prices. In addition, a corresponding increase from 0.8 to 1.7 million tons of FM was used in aquatic feed formulation within the same period [4]. Despite global FM production achieving a persistently high level, it cannot sufficiently accommodate aquatic farming's fast growth [5]. FM's high expense and erratic availability impede fulfilling the escalating production needs in the aquafeed sector [6]. Increasingly, alternative FM protein sources are gaining interest [7]. To mitigate the elevated cost of FM and curtail feed expenses, cost-effective and accessible agricultural by-products, including cottonseed meal (CSM) and blood meal (BM), can be integrated into tilapia diets in lieu of FM.

Cottonseed is an important plant protein source for animals. Due to its wide availability and cost-effectiveness compared with animal proteins, it is widely used as a protein nutrient worldwide [8–10]. Cotton meal is rich in high-quality plant protein and provides the main source of plant protein for fish [11]. However, it has gossypol, which is an antinutritional factor that is toxic to fish [12, 13]. Numerous studies indicate that fish feed CSM dosage largely hinges upon dietary-free gossypol and available lysine content [11]. Bound gossypol exhibits slow absorption, potentially inhibiting lysine intake, thereby inducing a risk of lysine deficiency [14]. BM, a slaughterhouse by-product, serves as a protein supplement for non-ruminant and ruminant feed [15]. BM is very rich in iron, which plays a good role in erythropoiesis, and iron from ferrous sulfate has been known to counteract the effect of gossypol from CSM in tilapia feed [16]. Moreover, BM possesses high lysine levels, serving as a significant source of important amino acids such as arginine, methionine, cystine, and leucine [17]. Specifically, BM effectively addresses lysine and methionine deficits in plant-based diets [18]. BM is rich in both iron and lysine, whereas CSM is rich in proteins and amino acid profile, though deficient in lysine. Besides, both ingredients are cheap and affordable and can be used to produce cost-effective tilapia feed.

Tilapia, specifically Nile tilapia (*Oreochromis niloticus*), is a common globally cultivated fish within its genus, projected to expand by nearly 60% from 4.3 to 7.3 million tons by 2030 [19]. Tilapia is quite resistant to stress and disease conditions, it produces white meat that is enriched with protein, lipids, and highly unsaturated fatty acids, which is nutritious and palatable with a broad market base. For profitability, a cost-effective diet for this species is essential to create. A lot of information is available on the use of CSM and BM, either single or in combination with other protein sources, to replace FM in tilapia feed, with encouraging results [20–22]. However, there have been limited studies on FM replacements with CSM and BM combination in tilapia. Therefore, this study intends to take advantage of the attributes of both protein by-products in combination to produce a cost-effective tilapia diet with CSM and BM combination supplementation in the GIFT tilapia juveniles diet. It aims to test to what extent this blend-in replacement of FM can improve growth performance and have no adverse effects on GIFT tilapia juveniles.

## 2. Materials and Methods

### 2.1. Diet Preparation and Experimental Design

Five isonitrogenous (31.5%) and lipidic (7.45%) diets were compounded for the trial with graded levels of a blend of CSM and BM in replacement of 0% (D-0, control group), 25% (D-25), 50% (D-50), 75% (D-75), 100% (D-100) FM (Table 1). Each of the five trials had three replicates randomly. Group one (D-0) was fed the control diet containing 16% FM, 1% BM, and 20% CSM. In other groups, changes in FM (12%, 8%, 4%, 0%), BM (3%, 5%, 7%, 9%), and CSM (23%, 26%, 29%, 32%) were made for group two (D-25), group three (D-50), group four (D-75), and group five (D-100), respectively. To prepare the experimental diets, feed ingredients were well grounded and sieved using a 0.2 mm size. Oil and water were sprayed into the mixture. Subsequently, the final blend was compacted into pellets (1.5 mm diameter) via a Lab pelletizer (Y90L-2, Xinchang Chenshi Machinery Co., Ltd., Zhejiang, China). The pellets were dried to ~10% moisture content under ambient conditions. Then, they were securely packaged in labeled plastic bags and refrigerated at −20°C for future utilization.

### 2.2. Experimental Fish and Management

GIFT tilapia juveniles were obtained from the Yixing research station of the Freshwater Fisheries Research Center, Chinese Academy of Fishery Sciences, Wuxi, Jiangsu, P. R. China. Fish were initially stocked in round fiberglass tanks (*φ*820 × 700 mm) and fed a commercial diet (No. 5271, 32% crude protein, Ningbo Tech-Bank Company Limited, Yuyao City, China) to acclimate for 2 weeks. After the acclimation, a total of 300 fish, weighing ~36.55 ± 0.15 g each, were equally distributed across 15 tanks, 3 tanks per group and 20 fish per tank. Fish received their respective meals thrice daily (7:30, 11:30, 16:30) over 60 days. Recycling water was extracted from subterranean sources, and a third of the tank's volume was refreshed weekly. All tanks were siphoned daily to remove feces. Water temp varied between 27.72 ± 0.45°C, pH level held steady at 7.64 ± 0.28, and dissolved oxygen concentration was noted to be 5.37 ± 0.11 mg/L during our experimental phase (YSI 650 MDS multi-probe system, YSI Inc. USA).

### 2.3. Sample Collection

Post 60 days rearing trial, all fish underwent a 24-h fast to empty their digestive tracts before sampling. A total of nine specimens per group (three fish from each tank) were anesthetized using MS-222 (100 mg/L) to collect samples. The blood was collected from caudal veins using heparinized hypo-dermic syringes (500 U sodium heparinized/mL). The blood sample was maintained at 4°C in nonheparinized tubes. Samples underwent further centrifugation for 10 min at 5000 rpm under identical conditions, and serum was extracted and preserved at −20°C for subsequent biochemical, hematological, and antioxidant enzyme analyses. Nine randomly selected fish from all tanks were weighed, and growth performance was calculated before sampling.

### 2.4. Growth Performance Parameters

• Weight gain rate (WGR, %) = [*W*1 – *W*0]/*W*0 × 100,
• Specific growth rate (SGR, %/day) = (In*W*1−In*W*0)/*t* × 100,
• Feed conversion ratio (FCR) = FI (g)/WG (g),
• Protein efficiency ratio (PER) = WG (g)/protein intake (g),
• Survival rate (SR, %) = 100 × (final fish number)/(initial fish number) × 100,
• Hepatosomatic index (HIS, %) = [liver weight (g)/W (g)] × 100,
• Viscerosomatic index (VSI, %) = [visceral weight (g)/W (g)] × 100,
• Condition factor (CF) = (*W*/*L*^3^) × 100,

where *W* is the body weight in (g), *W*1 is the final body weight (FBW), *W*0 is the initial body weight, *t* is the duration of the trial (in days), and *L* is the total length in centimeters.

### 2.5. Whole Body Composition Analysis

Analyses of whole-body proximate components (water, crude protein, crude fat, and ash) were conducted by the Association of Official Analytical Chemists guidelines [1]. Moisture was determined by oven drying at 105°C until constant weight. The Kjeldahl method was used to determine crude protein using an Auto Kjeldahl system (FOSS KT260, Switzerland). Crude fat content was evaluated using the ether extraction technique (Ankom, XT15 USA). Ash was derived via heating sample till incipient smoke was released and subsequent baking in an oven at 560°C for 5 h.

### 2.6. The Blood Parameters

White blood cell (WBC) count, red blood cell (RBC) count, hemoglobin (HGB), hematocrit (HCT), and platelet count (PLT) were quantified utilizing the BC-5300Vet Auto Hematology Analyzer, courtesy of Shenzhen Mindray Medical International Co., Ltd. (P.R. China). The levels of albumin (ALB), total cholesterol (TC), glucose (GLU), total protein (TP), aspartate transaminase (AST), triglyceride (TG), and alanine aminotransferase (ALT) in the plasma were assessed via Mindary BS-400 autoanalyzer employing kits procured from Shenzhen Mindary Bio-medical Electronics Co., Ltd., adhering to a previously established methodology.

Superoxide dismutase (SOD), glutathione-peroxidase (GSH-Px) activities, and malondialdehyde (MDA) levels were assayed through a spectrophotometer (UV-2100, Shanghai Jinhua Technology Instrument Co., Ltd., Shanghai, China) at 550, 412, 532 nm, respectively. Antioxidant-related parameter detection kits (SOD, GSH-Px, MDA) were bought from Nanjing Jiancheng Bioengineering Institute (Nanjing, China). Detailed methods can be found in the material methods of Zhou et al. [23].

### 2.7. Statistical Analysis

Statistical evaluation utilizing SPSS 20.0 revealed one-way ANOVA multiple tests, identifying significant trial variances via Duncan's multiple range test. Significance was set at *p* < 0.05. Outcomes are communicated in terms of mean ± standard error (M ± SE).

## 3. Results

### 3.1. Growth Performance and Feed Utilization

Table 2 summarized the impacts on growth due to varying percentages of FM substitution by CSM and BM in GIFT tilapia juveniles. With the increase of FM replacement by the CSM and BM, the FBW, SGR, and WGR were decreased and the FCR increased. FBW, WGR, SGR, and PER in the group of D-100 were significantly (*p* < 0.05) lower than the control group of D-0. Furthermore, FCR in the group of D-100 was higher than that of the control group of D-0 (*p* < 0.05). The viscerosomatic index increased to a plateau at 75% before a slight decline at 100%. The group of D-50, D-75, and D-100 of viscerosomatic index showed a significant increase (*p* < 0.05) compared to the control group of D-0, whereas no significant (*p* > 0.05) differences were observed in SR, CF, and FI of fish fed different dietary replacement levels.

### 3.2. Whole Body Composition

Whole-body compositions are presented in Table 3. FM replacements did not induce any significant effect (*p* > 0.05) on moisture content and crude lipid of whole-body compared to the control group. With the increase of FM replacement by the CSM and BM, both crude proteins and ash content were decreased, and crude protein and ash were significantly lower in the D-100 group compared to the control group (*p* < 0.05).

### 3.3. Serum Biochemical Parameters

Serum biochemical parameters are presented in Table 4. The concentration of GLU showed an increasing pattern with FM replacement level and reached the highest at 50% replacement (*p* < 0.05). The group of D-50 and D-75 of GLU showed a significant increase (*p* < 0.05) compared to the control group of D-0. With the rise in FM replacement by the CSM and BM, the TP in the group of D-100 (100% FM replacement) produced a significant reduced (*p* < 0.05) compared to the control. All treatments had no significant difference (*p* > 0.05) in ALB, TGs, and TC.

**Figure 1:**
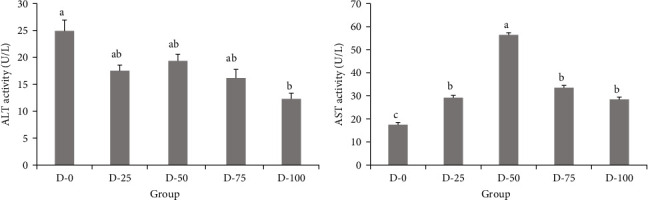
Serum ALT (A) and AST (B) activities in GIFT tilapia juveniles fed with a blend of cottonseed meal and blood meal supplemented in replacement of fish meal. Note: Values are presented as mean ± standard error (*n = 9*). Values with different superscripts in the same column are significantly different (*p* < 0.05). Fish replacement levels of 0%, 25%, 50%, 75%, and 100% represented D-0 (control group), D-25, D-50, D-75, and D-100 group, respectively.

ALT and AST activities are illustrated in Figure 1. Upon substitution, ALT exhibited a decreased trend with FM replacement, particularly significant for the D-100 group relative to the control (*p* < 0.05). However, AST activity increased from 25% to its peak at 50% FM replacement and then decreased to 100% FM replacement. The group of D-25, D-50, D-75, and D-100 AST activity was significantly higher (*p* < 0.05) than the control group of D-0.

### 3.4. Hematological Parameters

Hematological parameters are presented in Table 5. The WBCs showed significantly lower in the group of D-50, D-75, and D-100 than in the control group (*p* < 0.05). On the contrary, HCT was significantly lower in the D-50, D-75, and D-100 groups than in the control group, and RBC was significantly lower in the D-50 group than in the D-0, D-25, and D-100 groups (*p* < 0.05). There was no significant difference (*p* > 0.05) observed in the HGB and platelet between the treatment group and control group.

### 3.5. Plasma Antioxidant Enzymes

**Figure 2:**
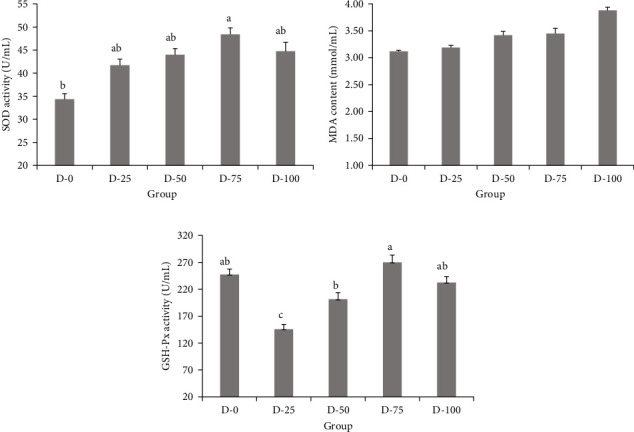
Serum SOD (A), MDA (B), and GSH-Px (C) in GIFT tilapia juveniles fed with a blend of cottonseed meal and blood meal supplemented in replacement of fish meal. Note: Values are presented as mean ± standard error (*n = 9*). Values with different superscripts in the same column are significantly different (*p* < 0.05). Fish replacement levels of 0%, 25%, 50%, 75%, and 100% represented D-0 (control group), D-25, D-50, D-75, and D-100 group, respectively.

Blood antioxidant enzymes are presented in Figure 2. It was observed that SOD activities rose from 25% to 100% FM replacement with no significant difference (*p* > 0.05) except 75% replacement. The data showed that SOD activity in the group of D-75 was significantly higher than that of the control group (*p* < 0.05). Furthermore, GSH-Px in the D-25 group was significantly lower than that in the control group and the experimental groups (*p* < 0.05), and there was no significant difference in MDA between the groups (*p* > 0.05).

## 4. Discussion

The feeding study showed no anomalies, with all dietary groups accepting their diets. The test diets had no significant effects on SRs. This was possible because the nutritional value of the diet was essential for fish growth. In this study, with the increase of FM replacement by the CSM and BM, the FBW, SGR, and WGR were decreased and FCR was increased. FBW, WGR, and SGR in the group of 100% FM replacement were lower than in the control group. Furthermore, FCR in the group of 100% FM replacement was higher than that of the control group. This indicated that 75% FM replacement (CSM 29% and BM 7% at FM 4% inclusion in diet) might be the best substitution level for GIFT tilapia. It also indicated that the gossypol in 75% of replacement diets exerted no visible adverse effects. Gossypol included in diets reduced growth and the intestinal amino acid absorption capacity of young grass carp [24]. It has been shown that ferrous ions in BM can neutralize the toxicity of cotton phenol in tilapia feeds [16]. And that further replacement of FM produced a decline in growth performance and feed utilization in terms of the above parameters. The decline in growth performance at 100% replacement could be a result of poor digestibility of BM (9% inclusion level) and this could affect the availability of lysine for absorption by fish. Similar to our results [25] found that 6% FM replacement with BM exerted no significant effect on FCR in pirarucu (*Arapaima gigas*), whereas 9% exhibited a significant effect on FCR. Besides, the other studies [26–28] also recorded similar results to this study, with inclusion levels of 60%–80% FM replacement producing no significant effect on growth performance and feed utilization.

In the present study, The D-100 group (100% FM replacement) significantly reduced crude protein and ash. Similar to Lovell's study, the high fiber in cotton meal reduces digestibility, which leads to reduced absorption, resulting in lower protein deposition and low mineral utilization, resulting in a lower proportion of ash in the fish [29]. Kumar et al. [2] reported that the high inclusion of CSM led to high contents of phytic acid, and this reduced the availability of minerals and proteins, thus leading to a subsequent deficiency in ash and lower crude protein content. This trend was similar to FBW, WGR, and SGR. It indicated that 75% FM replacement may be the best substitution level to attain 100% FM replacement by the combination of CSM and BM affected the utilization of dietary protein and led to the decrease of crude protein and ash content.

However, no substantial impact was observed on moisture or crude lipids relative to the control group, aligning with previous findings in catfish [30] and Japanese black sea bream [31]. Crude lipids were lower in the control to complete replacement, which is similar to research on Mohammadinafchi et al. [6]. According to the reference of Wang et al. [32], there was an inverse relationship between body moisture content and crude lipid. Further findings by Jiang et al. [33] revealed no negative impacts on body dry matter, protein, or lipid when incorporating 64% blends of CSM and soybean meal into Chinese mitten crab feed, substituting FM.

Plasma biochemical parameters aid in assessing fish's overall health [34]. In this study, dietary FM substitution by the combination of CSM and BM does not affect ALB, TG, and TC. Besides, similar results were found in Zhang's study, where there were no significant alterations in serum TG and TC levels after replacing FM with a protein by-product mixture [35]. In parrot fish, CSM replacement of FM was also found to significantly reduce serum TG and TC in the study [36]. A reason could be that the maximum inclusion level of 32% CSM in this study was not too high in the feed whereas they went up to 60.98% inclusion of CSM [37].

On the other hand, TP in this study experienced a significant effect at 100% FM replacement. Maybe this is because gossypol inhibited lysine absorption, which induced the lysine deficiency finally [14]. However, 75% FM replacement had no significant effect compared to the control; this may indicate that 75% FM replacement is the optimal replacement level for GIFT tilapia juveniles. In this study, BM is rich in both iron and lysine. Iron has been known to counteract the effect of gossypol from CSM in tilapia feed [16]. Besides, the concentration of GLU showed an increasing pattern with FM replacement level, and GLU was significantly higher in D50 and D75 than in controls. It indicated that 50% FM replacement might contribute to more energy in this fish species. Kavitha et al. [34] reported that GLU level was an important indicator of fish stress. However, it did not produce any adverse effects on fish [34]. However, Other studies have also shown that replacing FM with a mixture of wheat gluten and legume flour resulted in significant reductions in GLU and TC [38]. The difference among these studies could be attributed to the fact that we have applied BM and CSM whereas other studies have used other single or multiple protein sources in place of FM. The underlying mechanism needs further study.

ALT and AST are two enzymes that function importantly in metabolism [39]. The high levels of ALT and AST were biomarkers for liver damage [40]. In this study, activities of ALT displayed a decreasing pattern with the FM replacement. However, AST activities increased from 25% to their peak at 50% FM replacement and then decreased to 100% FM replacement [41]. It indicated that 75% replacement could reduce the damage to the liver of GIFT tilapia juveniles. The findings of this study were similar to the result of Wang et al. [40]. The significant decrease in ALT in the D-100 group may be due to the fact that although the fiber in a cotton meal may reduce feed utilization, the appropriate amount of fiber can promote intestinal peristalsis and the production of short-chain fatty acids, which can indirectly support the liver detoxification function and reduce ALT.

Blood parameters are extensively utilized for fish health assessment [42]. WBC plays a critical role in innate immunity against microbial infections. In the present study, the WBC was significantly lower in the group of 50%, 75%, and 100% FM replacement groups than in the control group, indicating that 50%–100% FM replacement affected the immunity of tilapia. HCT showed the same trend as WBC. Moreover, HGB and HCT declined from 50% to 100% replacement, which is in line with reports by Soltan [43], who observed that HCT and HGB concentrations in Nile tilapia significantly decreased proportionally with additional CSM inclusions and with the highest decline between 50% and 100% replacements. Based on HGB and HCT, the difference between these two studies lies in the fact that in this study the decrease in HGB was insignificant. The reason for this could probably be the complementary effect of BM since it is very rich in iron, which is a fundamental mineral needed for erythropoiesis [44].

In this study, SOD activities rose from 25% to 100% FM replacement and produced a significant difference at 75% replacement. An increase in SOD represents there is an improvement in the immune function of the fish, thus enabling it to better fight reactive oxygen species that can lead to oxidation in cell membranes. A significant increase in SOD activity was also found in Song's study after the replacement of FM by soy protein hydrolysate [45]. Furthermore, the concentrations of MDA and GSH-Px activities showed no significant change between the treatment group and the control group. These results were similar to Wang et al. [40], who noted that no significant effect was observed in the activity of MDA among treatments, whereas the activity of SOD increased significantly with increasing CSM inclusion level above 36%. This suggested that 75% FM replacement by the combination of CSM and BM in the diet might serve as an immunomodulatory to improve the immune ability of tilapia.

## 5. Conclusion

In conclusion, the optimum dietary CSM and BM level to replace an FM for GIFT tilapia juveniles was estimated to be 75% based on growth performance, whole body composition, blood cell counts, and serum biochemical indexes. This study provides a cost-effective way to replace an FM with the combination of CSM and BM in GIFT tilapia (*O. niloticus*) juveniles, it may provide some insights for the aquaculture industry.

## Figures and Tables

**Table 1:** Composition of experimental diet (%).

Ingredients	Dietary fish meal replacement				
	D-0	D-25	D-50	D-75	D-100
Fish meal^a^	16.0	12.0	8.0	4.0	0.0
Blood meal^b^	1.0	3.0	5.0	7.0	9.0
Cottonseed meal^c^	20.0	23.0	26.0	29.0	32.0
Soybean meal^d^	15.0	15.0	15.0	15.0	15.0
Wheat bran	25.0	23.0	21.0	19.0	17.0
Rice bran	15.4	15.4	15.4	15.4	15.4
Soybean oil	2.6	3.0	3.4	3.8	4.2
Vitamin premix^e^	1.5	1.5	1.5	1.5	1.5
Mineral premix^f^	1.5	1.5	1.5	1.5	1.5
CaH_2_PO_4_	2.0	2.0	2.0	2.0	2.0
Methionine	0	0.05	0.10	0.15	0.20
Attapulgite	0	0.55	1.10	1.65	2.20
Total	100	100	100	100	100
Proximate analysis					
Crude protein (%)	31.49	31.48	31.47	31.46	31.45
Crude lipid (%)	7.43	7.44	7.45	7.47	7.48
Nitrogen-free extract (%)	35.08	34.24	33.41	32.57	31.73
Ash (%)	10.98	11.24	11.51	11.77	12.04
Phosphorus (%)	1.32	1.21	1.11	1.01	0.91
Lysine (%)	1.79	1.78	1.76	1.75	1.74
Methionine (%)	0.60	0.60	0.61	0.61	0.61
Threonine (%)	1.16	1.16	1.14	1.12	1.10
Arginine (%)	2.49	2.53	2.58	2.63	2.68
Iron (mg/kg)	221.95	254.96	287.97	320.98	353.99

^a^Fish meal (CP 65%), provided by Coprinca Ltd. (Lima, Peru).

^b^Blood meal (CP 82%), provided by Weifang Meibaolai Animal Protein Co., Ltd. (Weifang, China).

^c^Cottonseed meal (CP 41%), supplied by Cargill (Shanghai, China).

^d^Soybean meal (CP 44%), supplied by Cargill (Shanghai, China).

^e^Vitamin mix (IU or mg kg^−1^ of diet): vitamin D, 250,000 IU; vitamin E, 4500 mg; vitamin K3, 220 mg; vitamin B1, 320 mg; vitamin B2, 1090 mg; vitamin B5, 2000 mg; vitamin B6, 5000 mg; vitamin B12, 116 mg; pantothenate, 1000 mg; folic acid, 165 mg; choline, 60,000 mg; biotin, 50 mg; niacin acid, 2500 mg; vitamin C, 2000 mg (provided by Wuxi Hanove Animal Health Products Co., Ltd. [Jiangsu, China]).

^f^Mineral mix (g kg^−1^ of diet); CuSO_4_·5H_2_O, 2.5 g; FeSO_4_·7H_2_O, 28 g; ZnSO_4_·7H_2_O, 22 g; MnSO_4_·4H_2_O, 9 g; Na_2_SeO_3_, 0.045 g; KI, 0.026 g; CoCl_2_·6H_2_O, 0.1 g (provided by Wuxi Hanover Animal Health Products Co., Ltd. [Jiangsu, China]).

**Table 2:** Growth performance and feed utilization in GIFT tilapia juveniles fed with a blend of cottonseed meal and blood meal supplemented in replacement of fish meal.

Parameter	Fish meal replacements				
	D-0	D-25	D-50	D-75	D-100
Initial body weight (g)	36.57 ± 0.08	36.50 ± 0.05	36.52 ± 0.06	36.52 ± 0.06	36.50 ± 0.08
Feed intake (g)	113.74 ± 0.66	113.67 ± 0.08	113.36 ± 0.82	113.55 ± 0.50	113.51 ± 0.24
Final body weight (g)	122.05 ± 2.19^a^	120.34 ± 0.27^ab^	118.18 ± 1.26^ab^	118.18 ± 1.22^ab^	116.46 ± 1.43^b^
Weight gain rate (%)	233.81 ± 6.70^a^	229.70 ± 0.80^ab^	223.63 ± 3.86^ab^	222.74 ± 3.09^ab^	219.05 ± 3.66^b^
Feed conversion ratio (%)	1.33 ± 0.03^b^	1.36 ± 0.01^ab^	1.38 ± 0.03^ab^	1.39 ± 0.02^ab^	1.42 ± 0.03^a^
Protein efficiency ratio (%)	2.71 ± 0.07^a^	2.66 ± 0.01^ab^	2.59 ± 0.04^ab^	2.58 ± 0.04^ab^	2.54 ± 0.02^b^
Specific growth rate (%)	5.24 ± 0.09^a^	5.19 ± 0.01^ab^	5.11 ± 0.09^ab^	5.09 ± 0.04^ab^	5.04 ± 0.05^b^
Condition factor (%)	3.18 ± 0.12	3.17 ± 0.15	3.40 ± 0.08	3.44 ± 0.08	3.30 ± 0.07
Viscerosomatic index (%)	8.21 ± 0.53^c^	8.83 ± 0.46^c^	9.59 ± 0.33^ab^	10.09 ± 0.37^a^	9.68 ± 0.26^ab^
Survival rate (%)	100.00 ± 0.00	100.00 ± 0.00	95.00 ± 5.00	100.00 ± 0.00	98.33 ± 1.67

*Note:* Values are presented as mean ± standard error (*n* = 9), and values with different superscripts in the same row are significantly different (*p* < 0.05).

**Table 3:** Results of whole body composition analysis in GIFT tilapia juveniles fed with a blend of cottonseed meal and blood meal supplemented in replacement of fish meal.

Parameter	Fish meal replacements				
	D-0	D-25	D-50	D-75	D-100
Moisture	66.72 ± 1.87	67.28 ± 0.12	67.64 ± 0.88	68.56 ± 0.30	69.10 ± 1.12
Crude protein (%)	15.73 ± 0.71^a^	14.99 ± 0.32^ab^	14.61 ± 0.18^ab^	14.16 ± 0.46^ab^	14.06 ± 0.12^b^
Crude lipid (%)	10.87 ± 1.06	10.03 ± 0.35	10.04 ± 0.86	10.01 ± 0.43	9.88 ± 1.01
Ash (%)	4.82 ± 0.07^a^	4.50 ± 0.09^ab^	4.44 ± 0.14^ab^	4.36 ± 0.18^ab^	4.19 ± 0.07^b^

*Note:* Values are presented as mean ± standard error (*n* = 9), and values with different superscripts in the same row are significantly different (*p* < 0.05).

**Table 4:** Blood biochemical parameters in GIFT tilapia juveniles fed with a blend of cottonseed meal and blood meal supplemented in replacement of fish meal.

Parameter	Fish meal replacements				
	D-0	D-25	D-50	D-75	D-100
Albumen (g/L)	10.42 ± 0.69	9.33 ± 0.44	9.83 ± 0.46	10.03 ± 0.99	9.17 ± 0.81
Triglyceride (mmol/L)	2.15 ± 0.30	1.95 ± 0.19	1.96 ± 0.13	2.24 ± 0.18	2.23 ± 0.30
Total cholesterol (mmol/L)	2.29 ± 0.20	2.25 ± 0.25	2.10 ± 0.16	2.48 ± 0.16	2.23 ± 0.30
Glucose (mmol/L)	3.56 ± 0.53^c^	4.86 ± 0.41^abc^	6.75 ± 0.60^a^	5.79 ± 0.86^ab^	4.53 ± 0.49^bc^
Total protein (g/L)	20.27 ± 1.28^a^	19.32 ± 0.45^ab^	18.63 ± 1.06^ab^	18.53 ± 0.55^ab^	16.97 ± 0.65^b^

*Note:* Values are presented as mean ± standard error (*n* = 9), and values with different superscripts in the same row are significantly different (*p* < 0.05).

**Table 5:** The hematologic parameters in GIFT tilapia juveniles fed with a blend of cottonseed meal and blood meal supplemented in replacement of fish meal.

Parameter	Fish meal replacements				
	D-0	D-25	D-50	D-75	D-100
White blood cell (10^9^/L)	190.28 ± 12.53^a^	180.94 ± 2.49^a^	152.98 ± 10.82^c^	162.56 ± 7.46^b^	156.52 ± 3.48^c^
Red blood cell (10^12^/L)	2.06 ± 0.16^a^	2.06 ± 0.10^a^	1.66 ± 0.14^b^	1.89 ± 0.13^ab^	2.10 ± 0.08^a^
Hemoglobin (g/L)	122.00 ± 8.30	121.44 ± 5.16	104.71 ± 8.15	112.50 ± 4.39	113.89 ± 4.67
Hematocrit (%)	42.03 ± 3.15^a^	40.02 ± 1.30^ab^	31.99 ± 2.57^d^	33.08 ± 2.23^c^	33.76±1.32^c^
Platelet (10^9^/L)	18.43 ± 4.38	17.57 ± 1.77	21.25 ± 2.70	18.83 ± 1.70	19.00 ± 1.97

*Note:* Values are presented as mean ± standard error (*n* = 9), and values with different superscripts in the same row are significantly different (*p* < 0.05).

## Data Availability

The data supporting this study's findings are available from the corresponding author upon reasonable request.
